# Detection of cutaneous leishmaniasis in three communities of Oti Region, Ghana

**DOI:** 10.1371/journal.pntd.0009416

**Published:** 2021-05-24

**Authors:** Richard Akuffo, Carmen Sanchez, Carmen Chicharro, Eugenia Carrillo, Naiki Attram, Mba-Tihssommah Mosore, Clara Yeboah, Nana Konama Kotey, Daniel Boakye, Jose-Antonio Ruiz-Postigo, Javier Moreno, Michael Wilson, Bismark Sarfo, Francis Anto

**Affiliations:** 1 Noguchi Memorial Institute for Medical Research, University of Ghana, Accra, Ghana; 2 School of Public Health, University of Ghana, Accra, Ghana; 3 WHO Collaborating Center for Leishmaniasis, Instituto de Salud Carlos III, Madrid, Spain; 4 U.S. Naval Medical Research Unit No. 3, Ghana Detachment, Accra, Ghana; 5 National Yaws Control program, Ghana Health Service, Accra, Ghana; 6 Department of Control of Neglected Tropical Diseases, World Health Organization, Geneva, Switzerland; Academic Medical Center: Amsterdam UMC Locatie AMC, NETHERLANDS

## Abstract

**Background:**

Cutaneous leishmaniasis (CL) is the most common type of leishmaniasis, a neglected tropical disease caused by parasites of the genus *Leishmania*. In Ghana, some studies in the Volta region have detected *Leishmania* parasites among persons with skin ulcers.

**Methodology/Principal findings:**

Using a cross-sectional study design, the prevalence of CL in three communities of the Oti Region of Ghana was investigated. Demographic and epidemiological data were obtained by a structured interviewer administered questionnaire. A total of 426 (12.4%) out of 3,440 participants screened had at least one skin ulcer. Of 595 skin ulcers sampled and tested by PCR for *Leishmania* infection, 150 (25.2%) ulcers from 136 individuals tested positive, accounting for an overall CL prevalence of 31.9% among persons with skin ulcers. Individual community CL prevalence of 23.2%, 29.8%, and 36.8% was observed in Ashiabre, Keri, and Sibi Hilltop respectively among persons with skin ulcers.

**Conclusions/Significance:**

Confirmation of CL in the study area suggests an active cycle of transmission of *Leishmania* infection. The observation of skin ulcers which tested negative to *Leishmania* infection suggests a need to test for additional causes of skin ulcers such as *Treponema pallidum pertenue* and *Mycobacterium ulcerans* in the study area.

## Author summary

Cutaneous leishmaniasis (CL) is a neglected tropical disease caused by parasites of the genus *Leishmania* and is transmitted by various species of female sandflies. CL usually begins as painless nodules and is usually characterized by skin ulcers which may be single or multiple. Although ulcers due to CL are often self-healing, they may become painful and result in scarring after healing. Typically, CL occurs on exposed parts of the body such as the neck, limbs, and face, which may be easily accessible to sandflies. Although cases of CL had previously been reported in some parts of the Volta region of Ghana, no previous case of CL had been reported in the Oti region. This study was initiated following reports of skin ulcers which were suggestive of CL in some communities of the Oti region. This study confirmed *Leishmania* infection in 150(25.5%) out of 595 skin ulcer samples obtained from 426 study participants. Given that 445(74,8%) of the skin ulcers tested negative for *Leishmania* parasite suggests a need for investigation of additional causes of skin ulcers such as yaws and Buruli ulcer in the study area.

## Introduction

Cutaneous leishmaniasis (CL) is an important neglected tropical skin disease (skin NTD) of public health importance and is the commonest form of leishmaniasis, characterized by skin lesions which may result in ulcers, scars, disability and stigma [1,2]. Globally, it is estimated that between 0.7 to 1.3 million new cases of CL are reported annually [3].

A localized outbreak of skin ulcers suspected to be cases of CL was first reported in Ghana from the Ho municipality of the Volta Region in 1999 based on the identification of *Leishmania* amastigotes in some skin lesion biopsies [4]. Subsequent studies have identified *L*. *major*, uncharacterized *Leishmania* species, and recently, members of the *Leishmania enriettii* complex from suspected CL cases in the Ho municipality, suggesting a complex epidemiology of CL in the region [5–7].

Although the Oti region has been part of the Volta region until the year 2019, no previous confirmation of CL cases had been made there. This study was therefore initiated following reports of skin ulcers which were suggestive of CL in some communities of the Oti region, after leishmanin skin test (LST) had been conducted to establish *Leishmania* infection (reported elsewhere).

## Materials and methods

### Ethics statement

Ethical approval to conduct this study was obtained from the ethics review committee of the Ghana Health Service (GHS-ERC006/08/18). Written informed consent was obtained from all study participants. For participants under 18 years, written consent was obtained from a parent or guardian.

### Study design

This study was based on a cross-sectional study design approach. The study was conducted from October to December 2018 in three communities of the Oti region of Ghana having at least three suspected cases of active CL (ACL). A suspected ACL lesion was defined clinically as any open ulcer with diameter bigger than 5mm. Prevalence of CL among study participants with skin ulcers was investigated. Demographic and epidemiological data were obtained by a structured interviewer administered questionnaire.

### Study area

This study was conducted in the following three communities of the Oti region of Ghana: Ashiabre, Keri, and Sibi Hilltop. Ashiabre is in the Tutukpene sub-district of the Nkwanta South municipality while Keri is in the Keri sub-district of the municipality. Sibi Hilltop is in the Sibi sub-district of the Nkwanta North district of the region.

The population of Nkwanta South municipality is estimated to be 117,878 with males constituting 49.6% of the population. Covering a land area of approximately 2733 km^2^, the Nkwanta South municipality is located between latitudes 7^o^ 30’ and 8^o^ 45’ North and longitude 0^o^ 10’ and 0^o^ 45’East [8].

The population of the Nkwanta North district is estimated to be 64,553 with males constituting 50.2% of the population. The district is located between Latitude 7°30’N and 8°45’N and Longitude 0°10’W and 045’E. It shares boundaries with Nkwanta South municipality to the south, Nanumba South to the north, Republic of Togo to the east, and Kpandai District to the west [9].

### Inclusion criteria

Eligible study participants were residents in the study community for ≥ 12 months, aged between 2 to 65 years (inclusive).

### Sample size considerations

For active case detection, assuming a current CL prevalence (P) of 22.1% [4,10], Z^2^ = (1.96)^2^ for 95% confidence interval D^2^ = maximum 0.05, a minimum sample size (N) of 265 individuals was required for screening for active case detection using the formula:

N = ((Z)^2^P/D^2^) *(1-P)

### Selection of households for study inclusion

Using a sorted list of households, 200 households (with an average of 5–7 persons per household) were selected for study inclusion in each study community using a systematic sampling approach. For this study, a household was defined as a person or a group of persons, who live together in the same house or compound and share the same house-keeping arrangements. The head of each household was defined as a male or female member of the household recognised as such by the other household members. The head of a particular household is generally the person with economic and social responsibility for the household. As a result, household relationships were defined with reference the household head [11]. The community household list was obtained for each study community based on a household census. The number of households per study community determined by household census was 945, 795, and 1184 in Ashiabre, Keri, and Sibi Hilltop respectively.

A sampling interval I was determined, where I = N/n with N being the sum of individual households in the study community while n was the number of households to be selected. The I was rounded to 2 decimal places.

Using Microsoft excel, the RANDBETWEEN command was used to generate a random decimal integer R between 0 and 1 rounded up to two decimal points. The sequence of households that were selected in each study community were R*I, R*I + I, R*I +2*I, R*I +3*I,….R*I + (n^-1^)*I, each rounded up to the next highest whole number [12]. With the assistance of community-based volunteers, the selected households were identified after which all members of the selected households aged 2 to 65 years were invited to participate in the study, using a door-to-door invitation approach. Because the invitation to participate in the study was extended to households, a household was not included in the study if the household head declined to allow his or her household to participate in the study. However, the agreement of the household head did not make it compulsory for every household member of age 2 to 65 years to participate in the study. Each household member was given the opportunity to go through the informed consent process to decide whether they wish to participate or not.

### Sampling of suspected active cutaneous leishmaniasis (ACL) lesions (ulcers)

Each study participant was asked to disclose the occurrence of any skin ulcer(s) on their body. Interviewers also examined the exposed parts of participants body such as legs, arms, neck, and face to identify any suspected active CL (ACL) lesion(s). The location, size, and duration of each suspected ACL was documented. For each suspected ACL lesion, a non-invasive diagnostic sampling technique using sequential tape strips with a diameter of 22 mm (D-Squame, CuDerm Corporation, Texas, USA) was used to obtain samples for subsequent DNA isolation [13].

**Fig 1:**
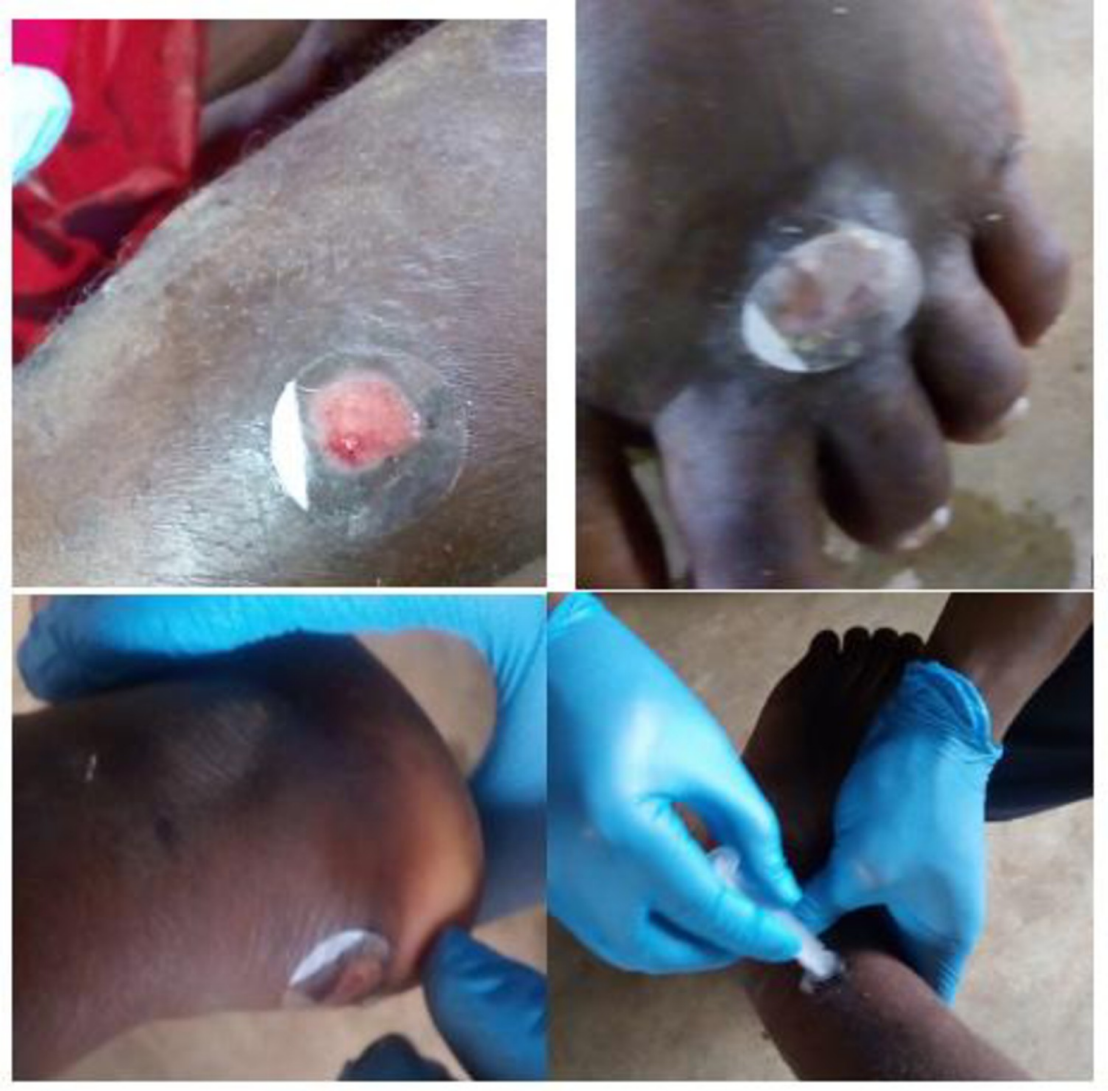
Non-invasive sampling of skin lesions.

For the non-invasive skin sampling, one tape disc was placed on each suspected skin lesion after which even pressure was applied to the disc on the lesion using a plunger which was gently held on the disc and pressed for approximately 20 seconds. The tape disc was then detached and transferred into a sterile 1.5ml Eppendorf vial and stored at 4°C for transportation to the laboratory for further analysis (Fig 1). Participants received standard wound care after sample collection.

### DNA isolation from tape strip disc and PCR amplification of Leishmania species

DNA extraction was performed using SpeedTools Tissue DNA Extraction Kit (Biotools, Inc).

A nested polymerase chain reaction (Ln-PCR) approach was used to amplify DNA of *Leishmania* species from the human skin lesions following an adaptation of the protocol by Cruz et al., 2002 [14], with the target being the small subunit ribosomal ribonucleic acid (SSU rRNA) gene. Positive control used was *Leishmania infantum* (JPC strain) with distilled water as negative control.

### Data management

Data was managed using Microsoft Access software version 2013 and analyzed using STATA software version 14. Association between nominal variables was assessed using Pearson’s chi square test of association and Fishers exact test. All statistical tests were performed at a 95% confidence level.

## Results

Of 600 households (200 in each study community) invited to participate in this study, a total of 587 households comprising 189 (32.2%), 200 (34.1%), and 198 (33.7%) from Ashiabre, Keri and Sibi Hilltop respectively, were included in this study. The study households had a total of 3718 members out of which 3,440 (92.5%) consisting of 1,194, 941, 1305 from Ashiabre, Keri, and Sibi Hilltop respectively were enrolled in the study.

The average household size was 6.3 with a range of 1 to 18 household members. Ashiabre and Sibi Hilltop had an average household size of 7 while Keri had an average household size of 5.

Out of 3440 persons physically examined for ulcers, a total of 595 skin ulcers were observed on 426 (12.4%) (Table 1). Of the 426 persons, 314 (73.7%) were within the age group 5–15 years while those under five constituted 13.6%. The number of skin ulcers observed on the participants ranged from 1 to 7 with those having one ulcer (47.1%) and two ulcers (27.6%) being the majority. Although skin ulcers were observed on various parts of the participants’ body, majority occurred on the lower legs (71.3%) and feet (17.1%). In Ashiabre, Keri, and Sibi Hilltop, 65.2%, 70.1%, and 74.3% of persons with skin ulcers had the ulcer on their lower legs respectively (Table 1).

**Table 1:** Individuals with skin ulcers, ulcers sampled and result of *Leishmania* PCR test.

Characteristic	Category	Ashiabre		Keri		Sibi Hilltop		Total		
		n	%	n	%	n	%	n	%	P value
Age of individuals with skin ulcers										
	<5 years	12	21.4	22	11.7	24	13.2	58	13.6	0.141
	5–15 years	35	62.5	145	77.1	134	73.6	314	73.7	
	16–45 years	9	16.1	19	10.1	18	9.9	46	10.8	
	>45 years	0	0	2	1.1	6	3.3	8	1.9	
	Total	56	100	188	100	182	100	426	100	
Sex of individuals with skin ulcers										
	Male	36	64.3	109	58	110	60.4	255	59.9	0.684
	Female	20	35.7	79	42	72	39.6	171	40.1	
	Total	56	100	188	100	182	100	426	100	
Number of Skin ulcers tested										
	1	31	44.9	116	41.3	133	54.3	280	47.1	<0.001
	2	28	40.6	84	29.9	52	21.2	164	27.6	
	3	8	11.6	46	16.4	37	15.1	91	15.3	
	4	0	0	30	10.7	11	4.5	41	6.9	
	5	2	2.9	5	1.8	0	0	7	1.2	
	6	0	0	0	0	5	2	5	0.8	
	7	0	0	0	0	7	2.9	7	1.2	
	Total	69	100	281	100	245	100	595	100	
Skin ulcer locations										
	Face/Head	3	4.3	5	1.8	6	2.4	14	2.4	0.029
	Upper arm	0	0	2	0.7	0	0	2	0.3	
	Lower arm	1	1.4	13	4.6	11	4.5	25	4.2	
	Palm/Back of palm	0	0	2	0.7	3	1.2	5	0.8	
	Chest	0	0	1	0.4	0	0	1	0.2	
	Back (upper part below neck))	0	0	0	0	2	0.8	2	0.3	
	Stomach	2	2.9	0	0	0	0	2	0.3	
	Buttocks	1	1.4	0	0	2	0.8	3	0.5	
	Thighs	1	1.4	8	2.8	6	2.4	15	2.5	
	Lower legs(crus/cnemis)	45	65.2	197	70.1	182	74.3	424	71.3	
	Feet	16	23.2	53	18.9	33	13.5	102	17.1	
	Total	69	100	281	100	245	100	595	100	
*Leishmania* pcr result										
	Negative	55	79.7	219	77.9	171	69.8	445	74.8	0.061
	Positive	14	20.3	62	22.1	74	30.2	150	25.2	
	Total	69	100	281	100	245	100	595	100	

PCR test of the 595 ulcer samples indicated that 150 (25.2%) of them were *Leishmania* positive. In the study communities, 14 (20.3%), 62 (22.1%), and 74 (30.2%) of skin ulcers tested from Ashiabre, Keri, and Sibi Hilltop respectively were positive for *Leishmania* (Table 1).

Of the 595 ulcer samples tested, 365 (61.3%) were obtained from males while 90 (60.0%) of the 150 *Leishmania* positive samples were also obtained from males. Also, 437 (73.4%) of the ulcer samples tested as well as 112 (74.7%) of the *Leishmania* positive ulcer samples were obtained from people within the age group 5–15 years (Table 2).

**Table 2:** Skin ulcers tested for *Leishmania* parasite using PCR by age and sex.

Sex	Age	Number of skin	*Leishmania* positive ulcers
		ulcers tested	n (%)
Males	< 5 years	48	8 (16.7)
	5–15 years	276	70 (25.4)
	16–45 years	36	10 (27.8)
	>45 years	5	2 (40.0)
	Subtotal	365	90 (24.7)
Females	< 5 years	45	13 (28.9)
	5–15 years	161	42 (26.1)
	16–45 years	19	4 (21.1)
	>45 years	5	1 (20.0)
	Subtotal	230	60 (26.1)
Total	< 5 years	93	21 (22.6)
	5–15 years	437	112 (25.6)
	16–45 years	55	14 (25.5)
	>45 years	10	3 (30.0)
	Total	595	150 (25.2)

The 150 *Leishmania* positive ulcer samples were obtained from 136 study participants of which 123 (90.4%) had single *Leishmania* positive skin ulcer, 12 (8.8%) had two *Leishmania* positive skin ulcers and 1 person had three *Leishmania* positive skin ulcers (Table 3). Majority of individuals with *Leishmania* positive ulcers were within the age group of 5–15 years (73.5%) followed by children under five (14.0%) and persons aged 16–45 years (10.3%). Across the study sites and among males and females respectively, majority of persons with *Leishmania* positive skin ulcer(s) were within the age group 5–15 years (Table 3).

**Table 3:** Distribution of individuals with *Leishmania* positive skin ulcers by age, sex, and community of residence.

Characteristic	Category	Ashiabre		Keri		Sibi Hilltop		Total		
		Male (%)	Female (%)	Male	Female	Male	Female	Male	Female	Total
Individuals with one *Leishmania* positive skin ulcer										
	<5 years	1 (25.0)	1 (12.5)	3 (9.4)	5 (27.8)	4 (9.8)	3 (15.0)	8 (10.4)	9 (19.6)	17 (13.8)
	5–15 years	3 (75.0)	6 (75.0)	23 (71.9)	11 (61.1)	31 (75.6)	15 (75.0)	57 (74.0)	32 (69.6)	89 (72.4)
	16–45 years	0	1 (12.5)	6 (18.8)	1 (5.6)	4 (9.8)	2 (10.0)	10 (13.0)	4 (8.7)	14 (11.4)
	>45 years	0	0	0 (0)	1 (5.6)	2 (4.9)	0	2 (2.6)	1 (2.2)	3 (2.4)
	Sub total	4 (100)	8 (100)	32 (100)	18 (100)	41 (100)	20 (100)	77 (100)	46 (100)	123 (100)
Individuals with two *Leishmania* positive skin ulcers										
	<5 years	0	0	0	1 (33.3)	0	1 (25.0)	0	2 (28.6)	2 (16.7)
	5–15 years	1 (100)	0	3 (100)	2 (66.7)	1 (100)	3 (75.0)	5 (100)	5 (71.4)	10 (83.3)
	16–45 years	0	0	0	0	0	0	0	0	0
	>45 years	0	0	0	0	0	0	0	0	0
	Sub total	1 (100)	0	3 (100)	3 (100)	1 (100)	4 (100)	5 (100)	7 (100)	12 (100)
Individuals with three *Leishmania* positive skin ulcers										
	<5 years	0	0	0	0	0	0	0	0	0
	5–15 years	0	0	0	0	1 (100.0)	0	1 (100.0)	0	1 (100.0)
	16–45 years	0	0	0	0	0	0	0	0	0
	>45 years	0	0	0	0	0	0	0	0	0
	Sub total	0	0	0	0	1 (100)	0	1 (100)	0	1 (100)
Individuals with *Leishmania* positive skin ulcer(s)										
	<5 years	1 (20.0)	1 (12.5)	3 (8.6)	6 (28.6)	4 (9.3)	4 (16.7)	8 (9.6)	11 (20.8)	19 (14.0)
	5–15 years	4 (80.0)	6 (75.0)	26 (74.3)	13 (61.9)	33 (76.7)	18 (75.0)	63 (75.9)	37 (69.8)	100 (73.5)
	16–45 years	0	1 (12.5)	6 (17.1)	1 (4.8)	4 (9.3)	2 (8.3)	10 (12.0)	4 (7.5)	14 (10.3)
	>45 years	0	0	0 (0)	1 (4.8)	2 (4.7)	0	2 (2.4)	1 (1.9)	3 (2.2)
	Sub total	5 (100.0)	8 (100.0)	35 (100)	21 (100)	43 (100)	24 (100)	83 (100)	53 (100)	136 (100)

The overall prevalence of cutaneous leishmaniasis (*Leishmania* infection observed among those with skin ulcers) was 31.9% (136/426) with prevalence of 23.2% (13/56), 29.8% (56/188), and 36.8% (67/182) observed in Ashiabre, Keri and Sibi Hilltop respectively.

**Fig 2:**
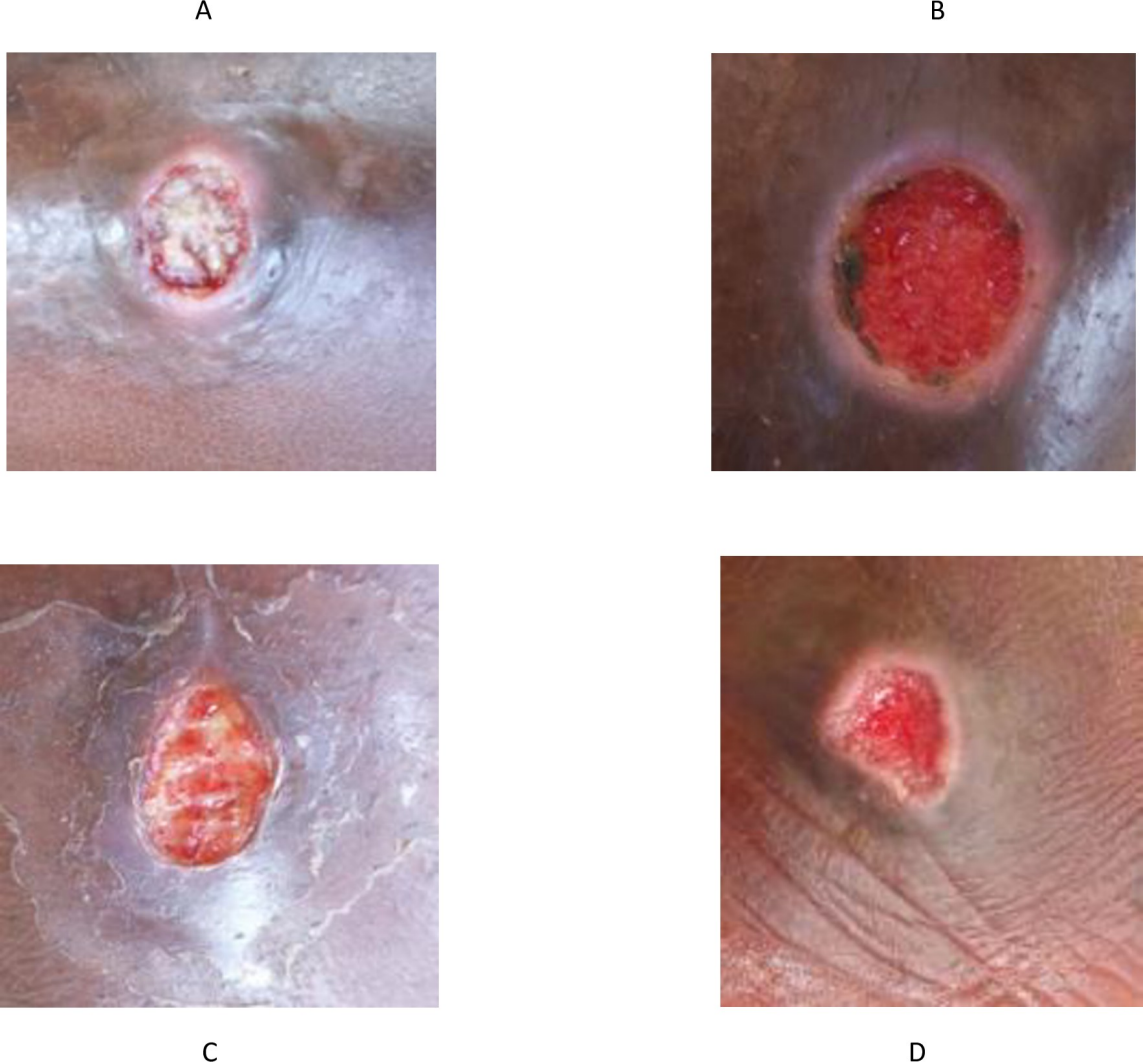
Examples of skin ulcers which tested positive for *Leishmania* parasite. A. Location: Left lower leg; dimension:10.1mm by 5.9mm. B. Location: Left lower arm; dimension:17.0mm by 15.1mm. C. Location: Left lower arm; dimension:17.6mm by 11.0mm. D. Location: Left lower leg; dimension:14.4mm by 5.2mm.

The average size of the skin ulcers observed was 10.2mm by 10.3mm with 573 (96.3%) of them reported to have started in the year 2018. Among the ulcers which started in the year 2018, 17 (3.0%) started between January to July 2018 while 13 (2.3%), 70 (12.2%), 346 (60.4%), 127 (22.2%) of them started in August, September, October and November of the year 2018 respectively. Examples of *Leishmania* positive skin ulcers observed is captured as Fig 2.

Of the 426 individuals with skin ulcers, 419 (98.4%) indicated that they applied some form of treatment. Majority of them (67.5%) used herbs while 35.3%, and 14.2% of them used hot stone and hot water respectively as treatment of their skin ulcers (Table 4).

**Table 4:** Summary of ulcer treatment methods reported by study participants.

Treatment	Ashiabre		Keri		Sibi Hilltop		Total	
method	No.	%	No.	%	No.	%	No.	%
Herbs	21	40.4	117	62.6	145	80.6	283	67.5
Hot stone	3	5.8	72	38.5	73	40.6	148	35.3
Dermacot	7	13.5	31	16.6	3	1.7	41	9.8
Penicillin	7	13.5	14	7.5	8	4.4	29	8.1
Amoxycillin	5	9.6	10	5.3	2	1.1	17	4.7
Hotwater	5	9.6	19	10.2	27	15	51	14.2
Other treatment	5	9.6	4	2.1	6	3.3	15	4.2
Total	52	100	187	100	180	100	419	100

## Discussion

### Cutaneous leishmaniasis among study participants

The control of CL requires an understanding of the disease epidemiology [15]. This study confirmed cutaneous leishmaniasis in the study communities by detecting *Leishmania* infection in 150 (25.2%) out of 595 ulcer biopsies tested by PCR. The overall prevalence of cutaneous leishmaniasis among persons with skin ulcers was 31.9% (136/426) with prevalence of 23.2% (13/56), 29.8% (56/188), and 36.8% (67/182) observed in Ashiabre, Keri and Sibi Hilltop respectively. In Mali, a systematic review reported a prevalence of 40.3% for cutaneous leishmaniasis among suspected CL cases[10].

Majority of the persons with CL in this study (73.5%) were in the age group of 5–15 years, with males in this age group constituting majority of those infected among persons with skin ulcers. A study in Mali which screened study participants with skin lesions for CL using PCR, confirmed *Leishmania* infection in samples from 8 persons who were all under 18 years [16].

A review of literature on CL suggests that although *Leishmania* infection and subsequent leishmaniasis disease generally tends to be influenced by factors associated with the host, the parasite, as well as the disease vectors, the prevalence of CL usually increases with age till about 15 years [17]. It is assumed that the prevalence of CL levels of at about 15 years because persons exposed early on in life to *Leishmania* infection may have acquired some level of immunity to the infection by then [17]. Observation of the highest prevalence of CL in 5–15 years age group in this study suggest a need to prioritize this group in future CL control planning in the study area.

### Treatment of persons with cutaneous leishmaniasis

An important aspect of disease control is treatment of affected people. The data on treatment of skin lesions by study participants indicate that majority of them use herbs (67.5%) followed by those who use hot stone (33.5%) and hot water (14.2%) respectively.

In the case of cutaneous leishmaniasis, the first choice of treatment is pentavalent antimonials with its attendant cost and possible adverse effects [18–22]. However, the evidence for what can be described as optimal treatment for CL has been described as patchy and generally weak. There is therefore a need for the development of improved guidelines for management of CL in addition to the conduct of more robust studies to improve the existing body of evidence for treatment of CL [18,23–25].

Furthermore, although efforts are ongoing to develop a vaccine against leishmaniasis, there is currently no vaccine licensed for use against leishmaniasis [26,27]. Given the gaps in the treatment of leishmaniasis and ongoing global efforts to develop vaccines, there is a need to develop measures in the local Ghanaian context, to protect people who are affected by leishmaniasis while research continues to provide data on critical aspects of the disease such as the vectors and reservoirs.

### Need for investigation of skin ulcers which were negative for *Leishmania* infection

Given that not all skin ulcers observed in the study communities were infected with *Leishmania* parasites, there is a need for continuous diagnoses of skin ulcers observed in the study communities in order to identify the ulcers infected by *Leishmania* parasite for the appropriate treatment to be applied [28–30].

Some studies have reported occurrence of other skin ulcers such as buruli ulcer, and yaws in Ghana [31–34]. A pilot study aimed at using azithromycin as treatment for yaws in some communities of the West Akim district of Ghana for instance, used sero-positivity based on a point of care dual treponemal and non-treponemal test as the primary outcome in addition to presentation with clinically active yaws like lesions (as secondary outcome) to select yaws cases [35].

As a result, future studies aimed at screening a larger sample of persons in the study area for yaws and other skin ulcer causing diseases such as buruli ulcer, incorporating more sophisticated laboratory diagnostic approaches may help to better characterize the causes of skin ulcers in the study area.

## Conclusions

Out of 426 individuals observed with various numbers of skin ulcers in the study communities, 136 (31.9%) individuals had various numbers of confirmed *Leishmania* positive skin ulcers. The observation of skin ulcers which tested negative to *Leishmania* infection suggests a need to test for additional causes of skin ulcers such as *Treponema pallidum pertenue* and *Mycobacterium ulcerans* in the study area.

### Limitations of the study

Molecular characterization of the ulcer samples for agents of other skin ulcer causing diseases reported in Ghana such as yaws, and buruli ulcer would have enriched the data.

Inclusion of a household in the study depended on the consent of the household head. This may have led to the exclusion of a few households, given that 587 households were included out of 600 households invited.

## Supporting information

S1 STROBE checklist Checklist according to The Strengthening the Reporting of Observational studies in Epidemiology (STROBE) guidelines. (DOCX)
