# Supplementary material for: Detection of cutaneous leishmaniasis in three communities of Oti Region, Ghana
Source: PLoS Negl Trop Dis. 2021 May 24;15(5):e0009416. doi: 10.1371/journal.pntd.0009416 (PMC8177633; doi:10.1371/journal.pntd.0009416)
Supplement: S1 STROBE checklist — (DOCX) [file pntd.0009416.s001.docx]

STROBE Statement— Detection of cutaneous leishmaniasis and treponemal infection in three communities of Oti Region, Ghana

|  | **Item No** | **Recommendation** | **Page No.** | **Relevant text from manuscript** |
| --- | --- | --- | --- | --- |
| **Title and abstract** | 1 | (*a*) Indicate the study’s design with a commonly used term in the title or the abstract | 2 | Line 43; Using a cross-sectional study design |
|  |  | (*b*) Provide in the abstract an informative and balanced summary of what was done and what was  found | 2 | Lines (41-56); Abstract |
| **Introduction** | | |  |  |
| Background/rationale | 2 | Explain the scientific background and rationale for the investigation being reported | 4 | Introduction (Lines 87-91) |
| Objectives | 3 | State specific objectives, including any prespecified hypotheses | 5 | Introduction Lines (93-98); This study was therefore initiated following reports of skin ulcers which were suggestive of CL in some communities of the Oti region, after leishmanin skin test (LST) had been conducted to establish *Leishmania* infection (reported elsewhere). |
| **Methods** | | |  |  |
| Study design | 4 | Present key elements of study design early in the paper | 5 | Methods, Lines (107-112); This study was based on a cross-sectional study design approach. |
| Setting | 5 | Describe the setting, locations, and relevant dates, including periods of recruitment, exposure, follow-up, and data collection | 5 | Methods, Lines (107 - 125); This study was conducted in the following three communities: Ashiabre, Keri, and Sibi Hilltop. Ashiabre is in the Tutukpene sub-district of the Nkwanta South municipality while Keri is in the Keri sub-district of the municipality |
| Participants | 6 | (*a*) *Cohort study*—Give the eligibility criteria, and the sources and methods of selection of participants. Describe methods of follow-up  *Case-control study*—Give the eligibility criteria, and the sources and methods of case |  |  |

|  |  | ascertainment and control selection. Give the rationale for the choice of cases and controls  *Cross-sectional study*—Give the eligibility criteria, and the sources and methods of selection of participants | 6 | Methods, Line 128 to 129; Eligible study participants were residents in the study community for > 12 months, aged between 2 to 65 years (inclusive). |
| --- | --- | --- | --- | --- |
|  |  | (*b*) *Cohort study*—For matched studies, give matching criteria and number of exposed and unexposed  *Case-control study*—For matched studies, give matching criteria and the number of controls per  case | N/A | This was a cross-sectional study |
| Variables | 7 | Clearly define all outcomes, exposures, predictors, potential confounders, and effect modifiers. Give diagnostic criteria, if applicable | 7-8 | Methods, Lines 158 - 177 |
| Data sources/ measurement | 8* | For each variable of interest, give sources of data and details of methods of assessment (measurement). Describe comparability of assessment methods if there is more than one group | 5 | Methods, Line 110 to 112; Prevalence of CL among study participants with skin ulcers was investigated. In a sub-sample of participants with ulcers, occurrence of treponemal infection was investigated. Demographic and epidemiological data were obtained by a structured interviewer administered questionnaire. |
| Bias | 9 | Describe any efforts to address potential sources of bias | 6 | Lines (145 to 146); A total of 200 households were selected from each study community for study inclusion using a systematic sampling approach described below. |

|  |  |  |  |  |
| --- | --- | --- | --- | --- |
| Study size | 10 | Explain how the study size was arrived at | 6. | Methods. Line 131 to 134;  a minimum sample size (N) of 265 individuals was required for screening for active case detection using the formula: |
| Quantitative variables | 11 | Explain how quantitative variables were handled in the analyses. If applicable, describe which groupings were chosen and why | 9 | Methods, Line 204 - 205;  Association between nominal variables was assessed using Pearson’s chi square test of association and Fishers exact test. All statistical tests were performed at a 95% confidence level. |
| Statistical methods | 12 | (*a*) Describe all statistical methods, including those used to control for confounding | 9 | Methods, Line 204 to 205; |
|  |  | (*b*) Describe any methods used to examine subgroups and interactions | N/A | There were no subgroups |
|  |  | (*c*) Explain how missing data were addressed | N/A | There was no missing data |
|  |  | (*d*) *Cohort study*—If applicable, explain how loss to follow-up was addressed  *Case-control study*—If applicable, explain how matching of cases and controls was addressed *Cross-sectional study*—If applicable, describe analytical methods taking account of sampling strategy | 7 | Methods: Lines 145-156); A total of 200 households were selected from each study community for study inclusion using a systematic sampling approach described below. |
|  |  | (*e*) Describe any sensitivity analyses | N/A |  |
| Results |  |  |  |  |
| Participants | 13* | (a) Report numbers of individuals at each stage of study—eg numbers potentially eligible,  examined for eligibility, confirmed eligible, included in the study, completing follow-up, and analysed | N/A | This was a cross-sectional study with only one stage |
|  |  | (b) Give reasons for non-participation at each stage | N/A |  |
|  |  | (c) Consider use of a flow diagram | N/A |  |
| Descriptive data | 14* | (a) Give characteristics of study participants (eg demographic, clinical, social) and information on exposures and potential confounders | 10 | Results, Line 215 - 229;  Of the 426 persons, 314 (73.7%) were within the age group 5-15 years while those under five constituted 13.6%. |

|  |  |  |  |  |
| --- | --- | --- | --- | --- |
|  |  | (b) Indicate number of participants with missing data for each variable of interest | N/A | There was no missing data |
|  |  | (c) *Cohort study*—Summarise follow-up time (eg, average and total amount) | N/A | This was a cross-sectional study |
| Outcome data | 15* | *Cohort study*—Report numbers of outcome events or summary measures over time | N/A | This was a cross-sectional study |
|  |  | *Case-control study—*Report numbers in each exposure category, or summary measures of exposure | N/A | This was a cross-sectional study |
|  |  | *Cross-sectional study—*Report numbers of outcome events or summary measures | 10-18. | Results, Line 213 to 304; |
| Main results | 16 | (*a*) Give unadjusted estimates and, if applicable, confounder-adjusted estimates and their precision (eg, 95% confidence interval). Make clear which confounders were adjusted for and why they were included | 12. | Results, Line 241 to 242. |
|  |  | (*b*) Report category boundaries when continuous variables were categorized | N/A | There were no continuous variables. |
|  |  | (*c*) If relevant, consider translating estimates of relative risk into absolute risk for a meaningful  time period | N/A | There were no estimates of relative risk |
| Other analyses | 17 | Report other analyses done—eg analyses of subgroups and interactions, and sensitivity analyses | N/A | There were no subgroups |
| **Discussion** |  |  |  |  |
| Key results | 18 | Summarise key results with reference to study objectives | 22. | Discussion, Line 308 to 371 |
| Limitations | 19 | Discuss limitations of the study, taking into account sources of potential bias or imprecision.  Discuss both direction and magnitude of any potential bias | 22 | Limitation, Line 379 to 381 |
| Interpretation | 20 | Give a cautious overall interpretation of results considering objectives, limitations, multiplicity of analyses, results from similar studies, and other relevant evidence | 22 | Conclusion, Line 373 to 377 |

| Generalisability | 21 | Discuss the generalisability (external validity) of the study results | 21 | Discussion, Line 368-369; |
| --- | --- | --- | --- | --- |
| **Other information** |  |  |  |  |
| Funding | 22 | Give the source of funding and the role of the funders for the present study and, if applicable, for the original study on which the present article is based | 23 | Lines 403-405; This project was funded by the post graduate training scheme fellowship in implementation science by the Special Programme for Research and Training in Tropical Diseases (WHO/TDR) at the School of Public Health, University of Ghana. The authors declare that the funder had no role in the study design, data collection, data analysis, data interpretation, and in writing the manuscript. |
